# Non-random sampling leads to biased estimates of transcriptome association

**DOI:** 10.1038/s41598-020-62575-x

**Published:** 2020-04-10

**Authors:** A. S. Foulkes, R. Balasubramanian, J. Qian, M. P. Reilly

**Affiliations:** 1 Massachusetts General Hospital, Harvard Medical School, Department of Medicine, Biostatistics, Boston, MA 02114 USA; 2 University of Massachusetts, Department of Biostatistics and Epidemiology, Amherst, MA 01003 USA; 3 Columbia University, Cardiology Division, Department of Medicine and the Irving Institute for Clinical and Translational Sciences, New York, NY 10032 USA

**Keywords:** Genetic association study, Biomarkers

## Abstract

Integration of independent data resources across -omics platforms offers transformative opportunity for novel clinical and biological discoveries. However, application of emerging analytic methods in the context of selection bias represents a noteworthy and pervasive challenge. We hypothesize that combining differentially selected samples for integrated transcriptome analysis will lead to bias in the estimated association between predicted expression and the trait. Our results are based on *in silico* investigations and a case example focused on body mass index across four well-described cohorts apparently derived from markedly different populations. Our findings suggest that integrative analysis can lead to substantial relative bias in the estimate of association between predicted expression and the trait. The average estimate of association ranged from 51.3% less than to 96.7% greater than the true value for the biased sampling scenarios considered, while the average error was − 2.7% for the unbiased scenario. The corresponding 95% confidence interval coverage rate ranged from 46.4% to 69.5% under biased sampling, and was equal to 75% for the unbiased scenario. Inverse probability weighting with observed and estimated weights is applied as one corrective measure and appears to reduce the bias and improve coverage. These results highlight a critical need to address selection bias in integrative analysis and to use caution in interpreting findings in the presence of different sampling mechanisms between groups.

## Introduction

The rapid emergence of large and publicly-available data resources across -omics platforms has fueled exponential interest in integrative analysis methods. These approaches involve combining data collected across independent groups of individuals to identify novel biological and clinical relationships. For example, there is an emerging literature on methods for unraveling the causal mechanisms of genetic associations with complex traits, and more specifically, for characterizing the mediating role of cell and tissue-specific gene expression in genetic associations, e.g.^1–8^. Many of these approaches involve leveraging and combining transcriptome-wide association studies (TWAS) and independent raw or summary-level genome-wide association studies (GWAS) data in a unified analysis framework. While the theoretical underpinnings of these approaches may be sound, their application to existing data resources requires careful consideration of the defining clinical and demographic characteristics of the cohorts being integrated.

We hypothesize that integrating data arising from two dissimilar populations can lead to substantial bias in estimates of association. In the application of two-stage least squares to transcriptomics data, an increasingly popular approach for this setting, this bias manifests in the estimates of association between genotype and expression and, in turn, the estimates of association between predicted expression and the trait. We evaluate the magnitude and direction of bias through an *in silico* case study in which data are derived from four established cohorts, namely: (1) Genome-Tissue Expression (GTEx) project cohort^9^ and independently generated data from (2) the National Health and Nutrition Examination Survey (NHANES)^10^, a population-based cohort; (3) the Chronic Renal Insufficiency Cohort (CRIC)^11^, an example “sick” cohort; and (4) the Genetics of Niacin and Endotoxemia (GENE) study cohort^12^, a representative “healthy” cohort.

The GTEx project data are derived from a convenience sample of cadavers, and as such, the anthropometric traits and related adipose gene expression may not be representative of a general US population, nor a chronic disease or very healthy population. The NHANES data, on the other hand, are a national representative sample of the general US population and therefore a referent population sample that is expected to be specifically generalizable. The CRIC data are additionally considered in our evaluation as a population-level disease sample enriched for multiple chronic diseases (including, obesity, hypertension, diabetes, chronic kidney disease, atherosclerosis and cardiovascular disease) that are common in the US and therefore of specific interest in complex disease gene discovery. Finally, the GENE data are derived from highly selected healthy and young volunteers and are of relevance in considering physiology and expression of optimal healthy states. These cohorts are chosen to reveal the range of potential bias in the estimates of association that can result from integrative analysis that disregards the heterogeneity between samples of individuals drawn from different populations.

A focus of this case study is the distribution of body mass index (BMI), a well-characterized quantitative trait with established heritability^13–15^ and also a known marker for multiple complex diseases and all cause mortality, e.g.^16–20^. Herein, BMI is used both as a surrogate measure of dissimilarly between cohorts and the primary phenotype under investigation. That is, we consider the setting in which the goal of analysis is to evaluate the mediating role of gene expression on the association between genotype and BMI through combining two independent samples. Our study investigates how the results of this analysis vary depending on the distributions of BMI in the two populations from which the independent samples are drawn. Simulations are performed to reflect known genotype-transcriptome and transcriptome-BMI associations as well as observed BMI distributions across established cohorts.

Selection bias refers to the situation in which the sampling mechanism results in an altered relationship between exposure and outcome^21^. Also referred to as biased sampling and ascertainment bias in some contexts, selection bias can result from the sampling mechanism systematically favoring features related to both the exposure and the outcome^22^. The impact of biases on -omics investigations is beginning to receive attention^23^, and an increasing body of literature exists on the resulting lack of transportability of GWAS findings^24,25^. Given the extensive and broad-based integration of GTEx cohort data in analysis pipelines – at present over 1100 PubMed Central citations – closer investigation into the possible implications of biased sampling in the generation of this cohort is warranted. Herein we consider the impact of selection bias on analysis involving integration of two independent data resources – specifically, the use of reference transciptome data to elucidate the biological mechanisms underpinning genetic associations with a quantitative trait, as described for example in^26^.

Inverse probability weighting (IPW) is applied (with known and unknown weights) as one potential corrective measure for this setting. IPW and covariate adjustments are well-described as the preferred approaches for addressing selection bias, e.g.^27–37^. These methods have been applied extensively and to a broad range of settings, including to address bias in the analysis of autopsy data^38^; however, to our knowledge, IPW has not been applied to integrated transcriptome analysis. While IPW as applied in our example appears to partially mitigate the bias observed in this setting, further work is needed to identify an optimal strategy. Our research aims to highlight this need by raising question about the validity of reported findings from application of integrative strategies without careful consideration of the representative-ness of cohorts across data resources.

## Results

### BMI distributions across cohorts

**Figure 1:**
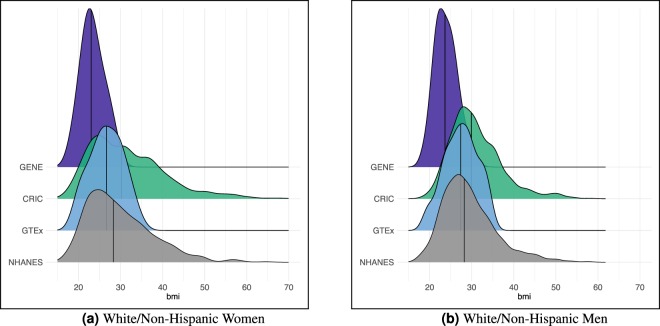
Estimated distributions of BMI by sex and cohort within White/non-Hispanics. (**a**) White/Non-Hispanic Women. (**b**) White/Non-Hispanic Men. The National Health and Nutrition Examination Survey (NHANES) data arise from a population-based cohort. Results are based on the 2015–2016 data and limited to individuals aged 21 to 70 for consistency with inclusion criteria for the Genome-Tissue Expression (GTEx) project cohort. The GTEx cohort is composed of deceased individuals. Ethnicity is not reported or unknown for 44.7% of this cohort. The results presented herein are based only on individuals recorded as White/Non-Hispanic. The Chronic Renal Insufficiency Cohort (CRIC) is a longitudinal study of individuals with chronic kidney disease; baseline data are reported and limited to individuals 21 to 70 years of age for consistency. The Genetics of Niacin and Endotoxemia (GENE) study cohort includes healthy adults aged 18 to 45. These results suggest that the cohorts represent samples of individuals from different underlying populations, which is further supported by the Kolmogorov-Smirnov and Wilcoxon rank sum tests in Table 1.

The distributions of BMI across cohorts are described and compared as one marker to indicate whether the cohorts were derived from similar populations. These results are stratified by sex and race/ethnicity because of the established modifying role of sex and race/ethnicity in genetic associations with BMI^39–41^ and limited to individuals age 21 to 70 for consistency across the GTEx, CRIC and NHANES cohorts. The estimated distributions of BMI by cohort and sex for White/non-Hispanic individuals are given in Fig. 1 and Table 1. As expected, the CRIC cohort exhibits the largest rightward skew in the BMI distribution for both women and men, with the percentages of women and men with BMI >30 *kg*/*m*^2^ equal to 51.0% and 49.7%, respectively. The GTEx cohort appears to be somewhat “healthier” than the NHANES cohort with a tighter BMI distribution and a slightly lower median value in both men and women. The distribution of BMI in the GENE cohort, with 72.5% and 67.6% of women and men, respectively, in the 18.5–24.9 *kg*/*m*^2^ range, is reflective of the relatively healthy group of young individuals selected for this study. Although sample sizes are limited, the results within Black/non-Hispanic women and men are consistent with these findings (Supplement Table S1 and Fig. S1).

**Table 1:** Summariy of BMI distributions by sex and cohort for White/non-Hispanic individuals age 21–70.

	Body Mass Index (*kg*∕*m*^2^, proportion by category)				KS^(*e*)^	Wilcoxon RS^(*f*)^
	< 18.5	18.5–24.9	25.0–29.9	≥30.0		
**NHANES**^(*a*)^						
Women (*n* = 679)	0.019	0.303	0.267	0.411	—	—
Men (*n* = 668)	0.012	0.254	0.356	0.377	—	—
**GTEx**^(*b*)^						
Women (*n* = 116)	0.000	0.353	0.440	0.207	< 0.001	< 0.001
Men (*n* = 211)	0.000	0.280	0.445	0.275	< 0.001	4.7 × 10^−3^
**CRIC**^(*c*)^						
Women (*n* = 588)	0.015	0.252	0.223	0.510	1.5 × 10^−3^	< 0.001
Men (*n* = 841)	0.001	0.141	0.360	0.497	< 0.001	0.001
**GENE**^(*d*)^						
Women (*n* = 91)	0.011	0.725	0.264	0.000	< 0.001	< 0.001
Men (*n* = 102)	0.000	0.676	0.314	0.010	< 0.001	< 0.001

^(*a*)^The National Health and Nutrition Examination Survey (NHANES) data arise from a population-based cohort. Results are based on the 2015-2016 data and limited to individuals aged 21 to 70 for consistency with inclusion criteria for GTEx.

^(*b*)^The Genome-Tissue Expression (GTEx) project cohort is composed of deceased individuals. Ethnicity is not reported or unknown for 44.7% of this cohort. The results presented herein are based only on individuals recorded as White/Non-Hispanic.

^(*c*)^The Chronic Renal Insufficiency Cohort (CRIC) is a longitudinal study of individuals with chronic kidney disease; baseline data are reported and limited to individuals 21 to 70 years of age for consistency.

^(*d*)^The Genetics of Niacin and Endotoxemia (GENE) study cohort includes healthy adults aged 18 to 45.

^(*e*, *f*)^Kolmogorov-Smirnov (KS) and Wilcoxon rank sum (RS) tests stratified by sex comparing the distribution of BMI in each each cohort to NHANES.

Kolmogorov-Smirnov (KS) and Wilcoxon rank sum (RS) tests are used to compare the distributions of BMI for each cohort to the NHANES cohort. The NHANES cohort is chosen as the referent group for these tests as it is a nationally representative sample, and therefore, a statistically significant test between a given cohort and NHANES suggests that the cohort is drawn from a population that differs from the general US population. P-values corresponding to each test stratified by sex are given in Table 1. In all cases, the KS test leads us to reject the null that the cohort is sampled from a population with the same BMI distribution as the NHANES data. Likewise, in all cases, the Willcoxon RS test rejects the null that the medians of the BMI distributions are equal. Again these results are consistent for Black/non-Hispanic women and men (Supplement Table S1) with the exception that we are unable to detect a difference in the BMI distribution for Black/Non-Hispanic men between the GTEx and NHANES cohorts.

### Simulation summary

The impact of biased sampling on integrated transcriptome analysis is evaluated through a simulation study that draws directly from the observed distributions of BMI across the four cohorts in Table 1. Population level data are simulated based on the distribution of BMI in Caucasian/non-Hispanic women in NHANES (see Methods). Four sampling scenarios are applied, as summarized in Table 2: (1) No selection bias: two random samples are selected from the simulated population cohort; (2) Selection bias in the TWAS cohort: the TWAS dataset is sampled from the simulated population cohort in a non-random fashion to mimic the BMI distribution observed in GTEx and the GWAS dataset is selected as a random sample; (3) Selection bias for both the TWAS and GWAS cohorts (case 1): the TWAS dataset is sampled in a non-random fashion to mimic the BMI distribution observed in GTEx and the GWAS dataset is sampled in a non-random fashion to mimic the BMI distribution in CRIC; and (4) Selection bias for both the TWAS and GWAS cohorts (case 2): the TWAS dataset is sampled in a non-random fashion to mimic the BMI distribution observed in GTEx and the GWAS dataset is sampled in a non-random fashion to mimic the BMI distribution in GENE. The four sampling scenarios are referred to respectively as: Random Sampling (RS); GTEx-RS; GTEx-CRIC; and GTEx-GENE.

**Table 2:** Simulations scenarios for evaluating impact of selection bias.

Scenario & Description	Source of sampling weights^(*a*)^		Abbreviation
	TWAS	GWAS	
(1) No selection bias	none^(*b*)^	none	Random Sampling (RS)
(2) Selection bias in TWAS	GTEx	none	GTEx-RS
(3) Selection bias in both TWAS and GWAS (case 1)	GTEx	CRIC	GTEx-CRIC
(4) Selection bias in both TWAS and GWAS (case 2)	GTEx	GENE	GTEx-GENE

^(*a*)^Data are generated by sampling from the observed NHANES BMI data using sampling weights based on the distribution of BMI in indicated dataset

^(*b*)^No sampling weights are used; the data are are sampled at random from the NHANES BMI data.

### Integrated transcriptome analysis with biased sampling

**Figure 2:**
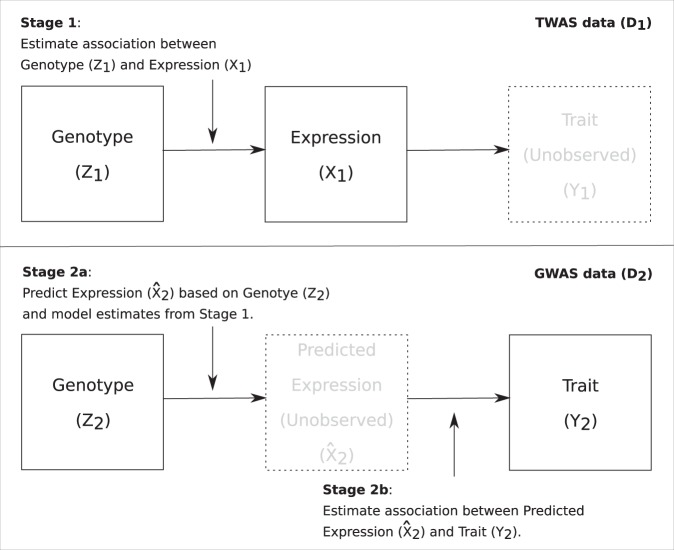
Two-stage least squares approach. Two-stage least squares (2SLS) is one established approach to evaluating the relationship between genetically regulated gene expression and a phenotype^26^. In the current study, we investigate how sampling of the TWAS (top panel) and GWAS (bottom panel) cohorts impacts the expression-trait association analysis. The observed data are defined as ${D}_{1}=\left\{({{\bf{z}}}_{i},{x}_{i}):i=1,\ldots ,{n}_{1}\right\}$ and ${D}_{2}=\left\{({{\bf{z}}}_{j},{y}_{j}):j={n}_{1}+1,\ldots ,n\right\}$, where **z**_*i*_, *x*_*i*_ and *y*_*i*_ are respectively, individual level genotype, expression and trait, and *n*_1_ and *n*_2_ are the sizes of two independent cohorts. We let *n*_1_ = 750 and *n*_2_ = 1500 which is consistent with the GTEx data and a small GWAS. In the first stage of the 2SLS analysis, a linear model is fitted using *D*_1_ by regressing *x*_*i*_ on **z**_*i*_. The estimated intercept, ${\hat{\alpha }}_{0}$, and coefficients, $\widehat{{\boldsymbol{\alpha }}}$, are recorded and using these estimates and *D*_2_ in the second stage, predicted expression is calculated as ${\widehat{x}}_{j}={\widehat{\alpha }}_{0}+{{\bf{z}}}_{j}\widehat{{\boldsymbol{\alpha }}}$. The association between predicted expression and the trait is then evaluated by regressing *y*_*j*_ on ${\widehat{x}}_{j}$, again based on a linear model with parameter estimates denoted ${\widehat{\gamma }}_{0}$ and ${\widehat{\gamma }}_{1}$.

A standard two-stage least squares (2SLS) approach^42^ is applied to evaluate the relationship between genetically regulated gene expression and a phenotype. The 2SLS approach is described for this setting in^26^ and summarized in Fig. 2. Briefly, the first stage of analysis in dataset 1 (TWAS) involves estimation of the association between genotype and expression. In the second stage in dataset 2 (GWAS), the association of predicted expression with a quantitative trait is estimated. Estimated bias in this expression-trait association of stage 2 is presented under a range of plausible sampling mechanisms for generating the TWAS and GWAS datasets.

**Figure 3:**
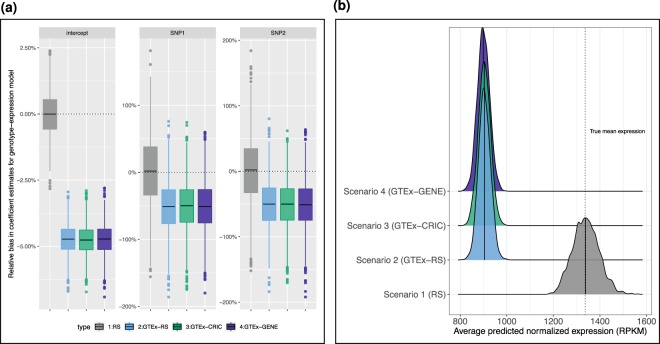
Results of first stage analysis with and without selection bias in sampling. (**a**) Relative bias $[100\ast (\widehat{\alpha }-\alpha )/\alpha ]$ in parameter estimates from eQTL analysis with (blue, green, purple) and without (grey) sampling bias. Results for all three biased sampling scenarios are shown for completeness; the same result is expected as the GTEx data distribution is used for sampling weights in all three scenarios. Biased sampling in this case study leads to under estimation of the stage one regression model parameters. (**b**) Distribution of average normalized predicted expression in GWAS sample based on stage 1 model fits with and without sampling bias. The dotted vertical line represents the population-level mean expression. The underestimation of model parameters in the biased sampling scenarios (Fig. 3a) leads to corresponding predictive distributions that are shifted downward with smaller variance compared to the RS scenario.

The results of the first stage analysis based on 2,000 simulations are provided in Fig. 3. The relative estimation bias, defined as the difference between the estimated and true parameter (for the genotype-expression association model) divided by the true parameter value and multiplied by 100 is provided in Fig. 3a. The RS scenario yields unbiased estimates, consistent with the extensive literature on maximum likelihood estimation, e.g.^43,44^. Results for all three biased sampling scenarios are included for completeness although the results are expected to be similar as these are the first stage modeling results. In all cases, the model intercept as well as both SNP level coefficients considered are biased downward. As shown in Fig. 3b, this results in underestimation of both the predicted expression and the prediction error for these three cases [scenario 2: mean = 906.4 Reads Per Kilobase Million (RPKM), standard deviation (sd) = 25.3; scenario 3: mean = 903.6 RPKM, sd = 26.0; scenario 4: mean = 900.2 RPKM, sd = 26.0]. The distribution of predicted expression for the RS case (mean = 1339.7 RPKM, sd = 56.1), on the other hand, is centered around the true population-level mean expression of 1336.4 RPKM.

**Figure 4:**
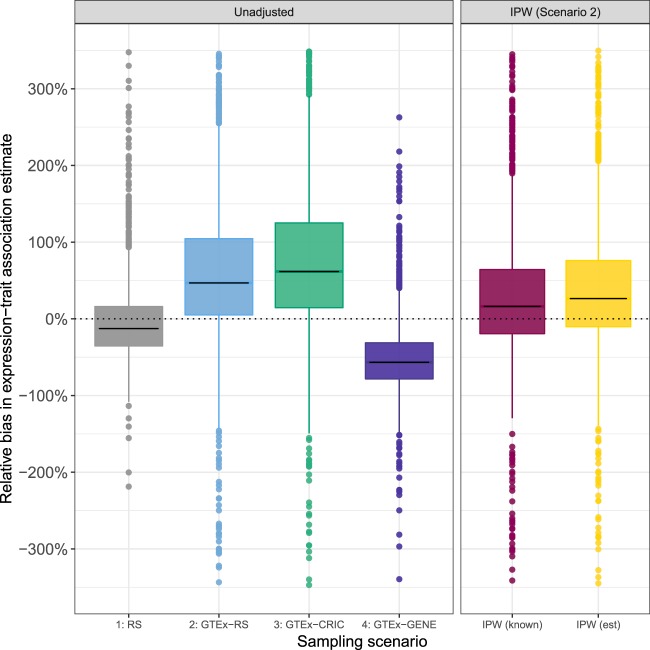
Relative bias in expression-trait association estimates obtained in the second stage, with and without selection bias in sampling. Relative bias $[100\ast ({\widehat{\gamma }}_{1}-{\gamma }_{1})/{\gamma }_{1}]$ in estimate of association between predicted expression and log BMI.This result suggests that the magnitude of bias can be large with average effects estimates as much 96.7% greater than the true value (median = 65.6%) as seen for scenario 3, and a high degree of variability across samples within a given scenario. Moreover, the direction of bias upward or downward depends on the sampling scheme. The percentage of data points outside of the visual range are <1%, 6.2%, 5.4%, <1%, 6.2% and 4.2%(from left to right). Application of IPW reduces the average relative bias that results from selective sampling from 73.6% (median = 50.7%) to 24.4% (median = 16.2%) and 44.5% (median = 27.2%), respectively, for known and estimated weights. Estimated weights are subject to error and as a result not expected to be as efficient as known weights.

The results of the second stage analysis are given in Table 3 and Fig. 4. Relative estimation bias is again defined as the ratio of the difference between the estimated and true parameter (in this case for the expression-BMI association) to the true parameter value and reported as a percentage. The estimate of association tends to be approximately unbiased for the first scenario with random selection [mean relative bias = −2.7%, median = −12.7, IQR = (−35.4, 16.1)]. This estimate tends to be biased upward for scenarios 2 [mean relative bias = 73.6%, median = 50.7, IQR = (4.3, 113.6)] and 3 [mean relative bias = 96.7%, median = 65.6%, IQR = (15.3, 135.9)] and biased downward for scenario 4 [mean relative bias = −51.3%, −56.7%, IQR = (−78, 4, −31.2)].

**Table 3:** Expression-trait association estimates obtained in the second stage, with and without selection bias.

	Average^(*a*)^			Coverage^(*d*)^
	Estimate^(*b*)^	Relative Bias^(*c*)^ (%)	95% CI length	
1: RS	0.146	− 2.7	0.167	0.750
2: GTEx-RS	0.260	73.6	0.385	0.695
3: GTEx-CRIC	0.295	96.7	0.398	0.631
4: GTEx-GENE	0.073	− 51.3	0.211	0.464
2: GTEx-RS + IPW (known)	0.187	24.4	0.297	0.751
2: GTEx-RS + IPW (estimated)	0.217	44.5	0.331	0.748

^(*a*)^Average is based on 2000 simulations.

^(*b*)^Estimate of predicted expression-trait association based on two stage regression imputation where the true population parameter for the observed expression-trait assocation is *γ*_1_ = 0.15.

^(*c*)^Relative bias is defined as $[100\ast ({\widehat{\gamma }}_{1}-{\gamma }_{1})/{\gamma }_{1}]$ where ${\widehat{\gamma }}_{1}$ is an estimate of association between predicted expression and the trait and *γ* is the true population parameter for the observed expression-trait association.

^(*d*)^Coverage is defined as the proportion of 95% CIs that cover the population-level expression-trait association parameter. This is expected to be less than 95% in scenario 1 as the prediction of expression in the two-stage regression imputation approach introduces measurement error.

Coverage defined as the percentage of simulations in which the 95% confidence interval for the expression-trait association parameter covers the true population level value, is estimated to be: 75.0%, 69.5%, 63.1% and 46.4% for the four respective sampling scenarios. Coverage is expected to be less than 95% even under settings of no sampling bias (RS), as 2SLS is based on single mean imputation, as described for example in^8^; however, this result indicates that selective sampling in the context of integrated analysis can further reduce coverage substantially.

### Application of inverse probability weighting (IPW)

As one corrective measure to address selective sampling, we apply inverse probability weighting (IPW)^27^ in the first-stage model fitting procedure using known and estimated sampling weights (see Methods) using data simulated according to scenario 2 (Table 2. IPW is an established approach for single cohort analysis to account for differences due to the non-random sampling from a target population, and involves applying a weight to each observation equal to the inverse of the probability that the observation was selected into the sample. Application of IPW partially mitigates the impact of biased sampling as the mean relative bias is reduced from 73.6% [median = 50.7%, IQR=(4.3, 113.6)] to 24.4% [median = 16.2%, IQR = (−20.4, 67.8)] and 44.5% [median = 27.2%, IQR = (−11.1, 80.8)] with known and estimated weights, respectively. The distributions of relative bias over the 2000 simulations using this corrective measure are illustrated Fig. 4b. Finally, the IPW coverage rates are 75.1% and 75.8% for known and estimated weights, respectively representing a marked improvement in coverage compared to scenario 2 without IPW (69.5%), and comparable to the RS scenario in which we see 75.0% coverage.

## Discussion

In summary, integrative analysis approaches that leverage independent data resources are increasingly popular as they offer substantial opportunity for novel discovery using existing datasets. However, consideration of fundamental design principles relating to sampling is imperative to ensure the validity and reproducibility of findings in this context. In the present investigation, *in silico* simulations that mirror characteristics of established cohorts arising from a range of different populations revealed the potential for systemic bias that can result from selective sampling. Importantly, as seen in the case studies, the magnitude of bias relative to the true parameter value for association between expression and a trait can be large, and the direction of this bias can be positive or negative, depending on the specific sampling mechanism. This degree of bias represents a grave matter, particularly in light of growing concerns over reproducibility and generalizability in genomics research. In practice, for most integrated -omics analysis involving independent datasets, the sampling mechanisms are not known although, in many cases, it will be clear that the populations from which the samples arise are dissimilar. Inverse probability weighting is one well-established approach used in single cohort analysis to address bias and our application suggests that it partially mitigates the errors introduced by selective sampling. In the case that the sampling weights are unknown, they can be estimated using observed covariate values. The precision of these estimates will impact the degree to which IPW attenuates the impact of sampling bias. Overall, this research suggest that judicious approaches to address pervasive sampling biases are critically required to ensure validity and generalizability of transcriptome association findings based on integrative analysis.

## Methods

### Datasets

The Centers for Disease Control and Prevention National Health and Nutrition Examination Survey (NHANES) data^10^ are designed to assess the health and nutritional status of adults and children in the United States through a combination of interviews and physical examinations. We use the NHANES 2015-2016 Demographics and Examination Data (https://wwwn.cdc.gov/nchs/nhanes/) with a combined dataset of *n* = 9544 individuals aged 0 to 80 years [51.0% Female, 49.0% Male; 21.5% Black/non-Hispanic, 30.9% Caucasian, 19.2% Mexican American, 12.9% other Hispanic, and 15.5% Other or Multi-Racial]. The median age is 27 [IQR = (9.0, 53.0)] and the median BMI is 25.20 kg/m^2^ [IQR = (19.90, 30.60)]. We limit this sample to include only White/non-Hispanic and Black/non-Hispanic individuals aged 21 − 70 years for consistency with the GTEx project data (see below). The NHANES data are used as the basis for our simulation as it is a sample that is expected to be representative of the general US population.

The Genotype-Tissue Expression (GTEx) project^9^ is an established and comprehensive public resource that includes whole genome sequencing (WGS) and cell and tissue-specific gene expression across 54 non-diseased sites. Our analysis is based on publicly available dbGaP Accession phs00424.v7.p2 data (https://www.gtexportal.org/home/datasets). The GTEx cohort is composed of *n* = 752 post-mortem donors aged 20 to 70 years [34% Female, 66% Male; 13% African American, 86% Caucasian, and 1% Other]. The median age is 56 [IQR = (47.0, 63.0)] and the median body mass index (BMI) is 27.26 kg/m^2^ [IQR = (24.28, 30.30)]. Information on exclusion criteria are described in^9^. Clinical conditions of relatively high prevalence in this cohort as compared to the US Adult population include Chronic Respiratory Disease or Chronic Lower Respiratory Disease (19.0%), Cerebrovascular Disease (9.6%), Ischemic Heart Disease (19.2%), Hypertension (55.5%), Renal Failure (13.4%) and Diabetes mellitus type II (21.8%). The heterogeneity of this cohort is further evidenced by the presence of conditions ranging from Schizophrenia (2.7%) and Major Depression (8.5%) to Arthritis (8.7%) and Pneumonia (9.6%). Individuals missing ethnicity (44.7%) are excluded from analysis.

The Chronic Renal Insufficiency Cohort (CRIC) Study is an ongoing observational study to characterize risk factors for progression of chronic kidney disease (CKD) and cardiovascular disease (CVD) among individuals with chronic renal insufficiency (CRI) (https://repository.niddk.nih.gov/studies/cric/). The cohort used for analysis is composed of n = 3939 individuals with CRI aged 21 to 75 years [45.1% Female, 54.9% Male; 41.6% White/non-Hispanic, 45.8% Black/non-Hispanic; 12.6% Other]. The median age is 59 years [IQR = (52, 66)], n = 1908 (48.4%) have diabetes mellitus, and the median BMI is 30.87 kg/m^2^ [IQR = (26.81, 36.09)]. We again limit analysis to individuals aged 21−70 years for consistency.

The Genetics of Evoked Response to Niacin and Endotoxemia (GENE) study is an NIH-sponsored investigation of the genomics of inflammatory and metabolic responses during low-grade endotoxemia^12,45,46^ in 294 healthy volunteers aged 18 to 45 years [51.4% Female, 48.6% Male; 65.6% White/non-HIspanic, 34.4% Black/non-Hispanic]. Participants were genotyped at baseline and multiple clinical variables including temperature and five plasma biomarkers were recorded repeatedly over 48 hour hospital visit after an endotoxin challenge. The median age is 24 [IQR = (21, 28)] and the median BMI is 23.32 kg/m^2^ [IQR = (21.70, 26.15)].

### Statistical approach

Data are simulated according to a composite model of association given by *y*_*i*_ = *γ*_0_ + *x*_*i*_*γ*_1_ + *ϵ*_*i*_ and *x*_*i*_ = *f*(**z**_*i*_) + *δ*_*i*_, where ${\epsilon }_{i} \sim N(0,{\sigma }_{\epsilon }^{2})$, ${\delta }_{i} \sim N(0,{\sigma }_{\delta }^{2})$, *ϵ*_*i*_⊥*δ*_*i*_, *y*_*i*_ is a quantitative trait, *x*_*i*_ is cell or tissue-specific expression for a single gene, and **z**_*i*_ is a vector of SNPs for individual *i* = 1, …, *n*. We let *f*(**z**_*i*_) be a linear function given by *α*_0_ + *z*_*i*1_*α*_1_ + *z*_*i*2_*α*_2_ with additive effects of each of two SNPs and minor allele frequencies of 0.20. Parameter values are selected to result in an average expression equal to the mean whole blood (WB) interleukin 1*β* (IL-1*β*) ${{\rm{\log }}}_{2}$ normalized expression in GTEx (*μ*_1_ = 10.39) and a distribution of natural log BMI that is consistent with the observed distribution for White/non-Hispanic women in the NHANES cohort (*μ*_2_ = 3.36): *α*_1_ = *α*_2_ = 0.06; *α*_0_ = *μ*_1_ − 0.4*(*α*_1_ + *α*_2_) = 10.23; ${\gamma }_{0}={\mu }_{2}-\bar{x}{\gamma }_{1}$; *γ*_1_ = 0.15; *σ*_*δ*_ = 1.6; and *σ*_*ϵ*_ = 0.065. A population of size *n* = 100, 000 is generated.

For the unbiased analysis (scenario 1), *n* = 2250 observations are sampled and randomly divided into two groups with *n*_1_ = 750 and *n*_2_ = 1500. For the biased sampling analysis (scenarios 2−4), *n*_1_ observations are sampled from the population with replacement using individual level sampling probabilities given by ${p}_{i}={w}_{i}/{\sum }_{i=1}^{{n}_{1}}{w}_{i}$ where *w*_*i*_ is defined as the proportion of GTEx observations that fall in the same decile of BMI distribution in the population as individual *i*. Additionally, for scenarios 3 and 4, *n*_2_ observations are sampled from CRIC and GENE respectively using similarly defined weights based on the corresponding cohorts. IPW is applied to scenario 2 using inverse probability weights for each individual equal to 1∕*p*_*i*_ where *p*_*i*_ is as defined above. IPW-known uses the true values of *p*_*i*_ used in sampling while IPW-estimated uses estimated values *p*_*i*_ based on predicted BMI. Estimates are derived by first fitting a model for BMI in the observed NHANES data with age and weight as predictor variables. A predicted BMI for each individuals in the GTEx data is then calculated based on the observed age and weight in GTEx and the fitted model from the NHANES data. Finally, inverse probability weight estimates are defined as above where predicted BMI values are used in place of observed values.

### Software

All analyses were performed using R version 3.5.2 (https://www.r-project.org/). Code and associated documentation is available at: https://github.com/andrea-foulkes/twas-transport.

## Supplementary information

Supplementary Information.
