# Supplementary material for: Non-random sampling leads to biased estimates of transcriptome association
Source: Sci Rep. 2020 Apr 10;10:6193. doi: 10.1038/s41598-020-62575-x (PMC7148323; doi:10.1038/s41598-020-62575-x)
Supplement: Supplementary file 1 — Supplementary Information. [file 41598_2020_62575_MOESM1_ESM.pdf]

Non-random sampling leads to biased estimates of transcriptome association

AS Foulkes[1,*], R Balasubramanian[2], J Qian[2], and MP Reilly[3]

[1]Massachusetts General Hospital, Harvard Medical School, Biostatistics Center, Boston, MA 02114 USA
[2]University of Massachusetts, Department of Biostatistics and Epidemiology, Amherst, MA 01003 USA
[3]Columbia University, Cardiology Division, Department of Medicine and the Irving Institute for Clinical and Translational Sciences, New York, NY USA 10025
[*]afoulkes@mgh.harvard.edu

ABSTRACT

Supplement

Supplement

Table S1. Distribution of BMI by cohort and sex for Black/Non-Hispanic individuals

	Body Mass Index (kg/m^2, proportion by category)					
	< 18.5	$18.5 - 24.9$	$25.0 - 29.9$	≥ 30.0	KS[(e)]	Wilcoxon RS[(f)]
**NHANES**[(a)]						
Women ($n = 541$)	0.015	0.196	0.232	0.557	–	–
Men ($n = 473$)	0.011	0.252	0.355	0.383	–	–
**GTEx**[(b)]						
Women ($n = 26$)	0.000	0.308	0.500	0.192	< 0.001	< 0.001
Men ($n = 43$)	0.000	0.256	0.488	0.256	0.090	0.164
**CRIC**[(c)]						
Women ($n = 767$)	0.009	0.124	0.166	0.701	< 0.001	< 0.001
Men ($n = 735$)	0.011	0.116	0.278	0.596	< 0.001	< 0.001
**GENE**[(d)]						
Women ($n = 60$)	0.017	0.617	0.367	0.000	< 0.001	< 0.001
Men ($n = 40$)	0.000	0.488	0.512	0.000	< 0.001	< 0.001

[(a)] The National Health and Nutrition Examination Survey (NHANES) data arise from a population-based cohort. Results are based on the 2015-2016 data and limited to individuals aged 21 to 70 for consistency with inclusion criteria for GTEx.
[(b)] The Genome-Tissue Expression (GTEx) project cohort is composed of deceased individuals. Ethnicity is not reported or unknown for 44.7% of this cohort. The results presented herein are based only on individuals recorded as Black/Non-Hispanic.
[(c)] The Chronic Renal Insufficiency Cohort (CRIC) is a longitudinal study of individuals with chronic kidney disease; baseline data are reported and limited to individuals 21 to 70 years of age for consistency.
[(d)] The Genetics of Niacin and Endotoxemia (GENE) study cohort includes healthy adults aged 18 to 45.
[(e,f)] Kolmogorov-Smirnov (KS) and Wilcoxon rank sum (RS) tests stratified by sex comparing the distribution of BMI in each each cohort to NHANES.

Figure S1. Distribution of BMI for Black/non-Hispanic individuals by sex and cohort

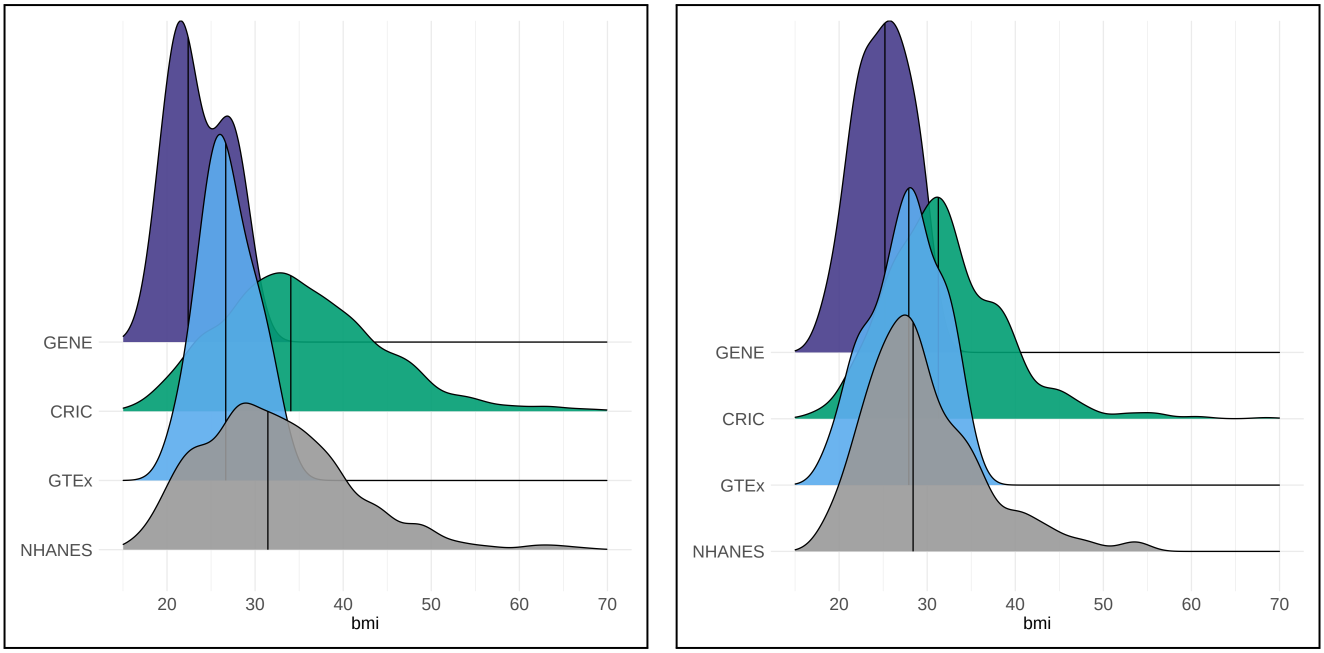

(a) Women **(b)** Men
